# Progress in eradication of HCV in HIV positive patients with significant liver fibrosis in Vienna

**DOI:** 10.1007/s00508-016-1162-y

**Published:** 2017-01-27

**Authors:** Sebastian Steiner, Theresa Bucsics, Philipp Schwabl, Mattias Mandorfer, Bernhard Scheiner, Maximilian Christopher Aichelburg, Katharina Grabmeier-Pfistershammer, Peter Ferenci, Michael Trauner, Markus Peck-Radosavljevic, Thomas Reiberger

**Affiliations:** 1 Division of Gastroenterology and Hepatology, Department of Internal Medicine III, Medical University of Vienna, Waehringer Guertel 18–20, 1090 Vienna, Austria; 2 Division of Immunology, Allergy and Infectious Diseases, Department of Dermatology, Medical University of Vienna, Vienna, Austria; 3 Vienna HIV & Liver Study Group, Vienna, Austria

**Keywords:** HIV, Hepatitis C virus, Sofosbuvir, Ledipasvir, 3D

## Abstract

**Aim:**

We aimed to investigate the efficacy of interferon and ribavirin-free sofosbuvir/ledipasvir (SOF/LDV) and ritonavir boosted paritaprevir/ombitasvir with or without dasabuvir (2D/3D) regimens in a real-life cohort of human immunodeficiency virus/hepatitis C virus (HIV/HCV) coinfected patients. The study focused on efficacy, need for changes in antiretroviral therapy (ART) due to drug-drug interaction (DDI), and treatment-associated changes in liver stiffness.

**Methods:**

In this study 36 patients (*n* = 21 SOF/LDV and *n* = 15 2D/3D) were retrospectively analyzed. Depending on the genotype the following treatment regimens were used: HCV genotype (GT)-1: either SOF/LDV or 3D, no patient with HCV-GT2 was included, HCV-GT3: SOF/LDV, HCV-GT4: 2D.

**Results:**

Approximately one third (35.3%) of patients were treatment-experienced and 13.9% had cirrhosis. Antiretroviral therapy had to be changed in 38.1% of SOF/LDV and 60% of 2D/3D patients prior to anti-HCV treatment due to expected DDIs. We observed sustained virologic response (SVR) rates of 100% in patients treated with SOF/LDV (19/19) and 2D/3D (14/14). One 2D/3D patient was lost to follow-up, while two SOF/LDV patients died during therapy from non-treatment-related causes. They were excluded from the analysis. Between baseline and follow-up liver stiffness decreased from 11.4 to 8.3 kPa (*p* = 0.008) and from 8.1 to 5.7 kPa (*p* = 0.001) in SOF/LDV and 2D/3D patients, respectively.

**Conclusions:**

We confirmed the excellent HCV eradication rates >95% in a real-life cohort of HIV/HCV coinfected patients treated with SOF/LDV and 2D/3D. We observed no HCV relapse or breakthrough. More patients treated with 2D/3D required a change in ART than patients treated with SOF/LDV. Additionally, HCV eradication led to a rapid decline in liver stiffness.

## Introduction

Chronic hepatitis C virus (HCV) infections are estimated to affect 0.3% of the overall population in Austria; however, only one third of patients are aware of their infection [1]. According to the Joint United Nations Programme on HIV/AIDS (UNAIDS) approximately 9000 people in Austria are living with human immunodeficiency virus (HIV) [2]. Of those approximately 20% are coinfected with HCV [3]. Liver-related mortality, mainly due to viral hepatitis, remains a major cause of death amongst HIV-positive individuals [4]. When compared with HCV mono-infected patients, HIV/HCV coinfected patients progress faster to cirrhosis [5] and show increased liver-related mortality [6, 7]. Since eradication of HCV improves overall survival among HIV/HCV coinfected patients [8], the European Association for the Study of the Liver (EASL) recommends prioritizing treatment of HCV in patients who are coinfected with HIV [9].

In 2008 there were an estimated 38,000 (range 8000–60,000) individuals with anti-HCV antibodies (anti-HCV prevalence rate of 0.5%; (0.1–0.7%)) [2, 26]. With a viremic rate of 73.9%, there were approximately 28,000 (range 6000–44,000) viremic individuals, corresponding to a viremic prevalence rate of 0.3% (0.1–0.5%).

In the era of pegylated interferon (IFN) and ribavirin (RBV), the majority of HIV/HCV coinfected patients remained untreated for HCV [10]. Patients with an urgent need for treatment due to established cirrhosis as well as those with psychiatric comorbidities were often not eligible for IFN-based therapy. Furthermore, even eligible patients refused anti-HCV therapy since adverse events (AEs) were common while the chance of cure was suboptimal [10–12].

Triple therapy with IFN/RBV plus one of the first generation direct acting antivirals (DAA) boceprevir [13] or telaprevir [14] achieved higher rates of sustained virologic response (SVR); however, their use was limited to HCV genotype (HCV-GT) 1 and treatment acceptance remained low due to contraindications, significant impairments of health-related quality of life during treatment [15] and patient refusal [16].

With the approval of second generation DAAs by the European Medicines Agency a new era of HCV therapy has started. Currently, IFN-free DAA combinations are only reimbursed by Austrian health insurance if a patient has significant liver fibrosis (METAVIR score ≥F2 or liver stiffness ≥7.0 kPa). Available and reimbursed second generation DAA regimens include sofosbuvir/ledipasvir (SOF/LDV) [17] and ritonavir boosted paritaprevir/ombitasvir with (3D) or without (2D) dasabuvir [18] which demonstrated excellent cure rates in clinical trials. In the ION-4 trial [17] SOF/LDV achieved an overall SVR rate of 96% in HIV/HCV coinfected patients with HCV-GT1 and HCV-GT4 treated for 12 weeks. In the ELECTRON-2 [19] trial SOF/LDV for 12 weeks achieved a SVR rate of only 64% in HCV-GT3 patients, while those who additionally received RBV had a SVR rate of 100%. In the ION-4 [17] trial SOF/LDV was well-tolerated with no discontinuation due to treatment-related AE.

In the TURQUOISE-1 [18] trial, 3D in combination with RBV achieved an SVR rate of 94% in HIV/HCV coinfected patients with HCV-GT1 treated for 12 weeks. The AE were rare and no patient discontinued treatment. The PEARL-1 [20] study investigated the use of 2D for HCV-GT4 and achieved SVR rates of 91% in non-cirrhotic, treatment-naïve HCV monoinfected patients; however, due to strict inclusion and exclusion criteria the majority of coinfected patients would not have been able to participate in the ION-4 and TURQUOISE-1 trials [21]. In these trials, patients were commonly excluded due to restrictions to specific antiretroviral therapies, active drug use, detectable HIV-RNA or due to low CD4 cell counts. Thus, it is uncertain whether these promising results can be extrapolated to real-life patients. Due to the addition of the ritonavir boosted protease-inhibitor paritaprevir, 2D/3D carries a substantial potential for drug-drug interactions (DDI) [22]. Although SOF/LDV moderately increases tenofovir disoproxil fumarate (TDF) levels similar to those seen when combining TDF with a ritonavir boosted HIV protease inhibitor (PI) [22], no dose adjustments or changes in antiretroviral therapy (ART) are necessary. In contrast, the use of 2D/3D in combination with HIV PI or non-nucleoside reverse transcriptase inhibitors (NNRTI) is not recommended due to DDI [23].

Liver stiffness measured by transient elastography is a surrogate marker of liver fibrosis [24] and portal hypertension [25] and predicts hepatic decompensation in HIV/HCV coinfected patients [26]. Interestingly, recent studies revealed that both IFN-based [27] and IFN-free therapies [28] improved liver stiffness in HIV/HCV coinfected patients, suggesting liver fibrosis regression and a reduction in portal pressure [29].

The real-life efficacy of SOF/LDV and 2D/3D has yet to be demonstrated in HIV/HCV coinfected patients [21]. Thus, we investigated the efficacy of IFN and RBV-free SOF/LDV and 2D/3D regimens in a real-life cohort of HIV/HCV coinfected patients. Moreover, we assessed the need for changes in ART due to DDI as well as the course of liver stiffness.

## Patients, materials and methods

### Study population

All HIV/HCV coinfected patients treated at the Medical University of Vienna with either SOF/LDV or 2D/3D who completed the SVR visit by July 2016 were retrospectively analyzed. Based on these criteria 36 patients were included: SOF/LDV *n* = 21 and 2D/3D *n* = 15.

### Assessed parameters

Epidemiological characteristics and HIV as well as HCV infection parameters were collected from patient medical history. HCV-GT was determined using the VERSANT HCV Genotype 2.0 Assay Line Probe Assay (LiPA) (Siemens Healthcare Diagnostics, Tarrytown, NY), while HCV-RNA was assessed using the Abbott RealTime HCV assay (Abbott Molecular, Des Plaines, IL) with a lower limit of quantification (LLQ) of 12 IU × ml^−1^.

### HIV therapy

Prior to HCV treatment initiation, ART was changed to two nucleoside reverse transcriptase inhibitors (NRTIs) combined with an integrase inhibitor (II) in the case of suspected DDIs, tolerance reasons, or for HIV treatment simplification (reduction of pill burden). HIV-RNA was assessed using the Roche COBAS® TqaMan HIV-1 Test, v2.0 (Roche, Vienna, Austria) with a LLQ of 20 copies × ml^−1^.

### HCV therapy

The decision on the therapy regimen was made based on HCV-GT and reimbursement by the Austrian health insurance. The following regimens were used: HCV-GT1: either SOF/LDV or 3D, no patient with HCV-GT2 was included, HCV-GT3: SOF/LDV, HCV-GT4: 2D. A total of 21 patients were treated with SOF/LDV (Harvoni® 400/90 mg, Gilead, Vienna, Austria) and 15 patients were treated with ombitasvir/paritaprevir/ritonavir (Viekirax® 12.5 mg/75 mg/50 mg, Abbvie, Vienna, Austria) two tablets once daily. In the case of HCV-GT1 infection, dasabuvir (Exviera® 250 mg, Abbvie, Vienna, Austria) twice daily was added. Treatment duration was 12 weeks, except for patients with cirrhosis or HCV-GT3 in whom the treatment duration was extended to 24 weeks. In HCV-GT3 patients with excellent virologic response treatment duration was shortened to 16 or 20 weeks; however, in some patients, treatment prolongation was denied by the Austrian health insurance. Sustained virologic response (SVR) was defined as a negative PCR result 12 weeks after cessation of treatment.

### Liver stiffness measurement

Liver stiffness was measured at baseline and follow-up 12 weeks after cessation of treatment via transient elastography (Fibroscan, Echosens, Paris, France), as previously described [24].

### Statistics

Analyses were performed using IBM SPSS Statistics 23 (SPSS, IBM, Armonk, NY) and GraphPad Prism 7 (GraphPad Software, La Jolla, CA). Continuous variables were reported as mean ± standard error of the mean, whereas categorical variables were reported as number and proportion of patients with the certain characteristic. Student’s t‑test was used for comparisons of continuous variables. Comparisons of categorical variables were performed using Fisher’s exact test. Paired t‑test was used for comparing baseline and follow-up liver stiffness. A *p*-value ≤0.05 was denoted statistically significant.

### Ethics

This study was conducted in accordance with the Declaration of Helsinki and approved by the local ethics committee of the Medical University of Vienna (No. 1814/2015).

## Results

### Patient characteristics

A full list of patient characteristic is given in (Table 1).

**Table 1:** Patient characteristics

	Overall	*SOF/LDV*	2D/3D	*p*-value
**Age (years)**	43.7 ± 2.8	45 ± 2.4	41.9 ± 3.2	0.419
**Sex**				
Male	26 (72.2%)	16 (76.2%)	10 (66.7%)	0.709
Female	10 (27.8%)	5 (23.8%)	5 (33.3%)	
**BMI**	24.4 ± 1.4	23.8 ± 1.4	25.3 ± 1.4	0.426
**History of alcohol abuse**	7 (19.4%)	4 (19%)	3 (20.0%)	1
**HCV infection parameters**				
Baseline HCV-RNA (log IU × ml^−1^)	6.12 ± 0.2	6.22 ± 0.16	5.98 ± 0.23	0.392
*HCV genotype*				
HCV-GT1	25 (69.4%)	13 (61.9%)	12 (80.0%)	n. a.
HCV-GT2	0	0	0	
HCV-GT3	8 (22.2%)	8 (38.1%)	0	
HCV-GT4	3 (8.3%)	0	3 (20.0%)	
*Transmission*				
IVDA	22 (61.1%)	13 (61.9%)	9 (60%)	n. a.
MSM	7 (19.4%)	4 (19.0%)	3 (20%)	
Heterosexual contact	4 (11.1%)	3 (14.3%)	1 (6.7%)	
Other	1 (2.8%)	0	1 (6.7%)	
Unknown	2 (5.6%)	1 (4.8%)	1 (6.7%)	
*Previous HCV treatment*	12 (35.3%)	6 (28.6%)	7 (46.7%)	0.462
**HIV infection parameters**				
Antiretroviral therapy	35 (97.2%)	20 (95.2%)	15 (100%)	1
HIV-RNA suppression at baseline	27 (84.4%)	15 (75%)	12 (100%)	0.130
CD4+ T lymphocyte nadir (cells/μL)	300 ± 62	293 ± 49	311 ± 79	0.879
CD4+ T lymphocytes (cells/μl)	654 ± 99	617 ± 85	699 ± 112	0.664
**Liver stiffness ≥7.1 kPa**	16 (44.4%)	8 (38.1%)	8 (53.3%)	0.310
**Liver stiffness ≥9.5 kPa**	5 (13.9%)	3 (14.3%)	2 (13.3%)	1
**Liver stiffness ≥12.5 kPa**	5 (13.9%)	4 (19.0%)	1 (6.7%)	0.376
**Portal hypertension ≥6 mm Hg**	6 (16.6%)	4 (19.0%)	2 (13.3%)	1
**Clinically significant portal hypertension ≥10 mm Hg**	3 (8.3%)	3 (14.3%)	0	0.250
**AST (IU/mL)**	72.03 ± 14.23	85.38 ± 16.62	53.33 ± 7.86	0.101
**ALT (IU/mL)**	108.33 ± 33.24	140.33 ± 40.61	63.53 ± 11.32	0.092
**GGT (IU/mL)**	148.61 ± 37.15	155.67 ± 41.07	138.73 ± 30.67	0.745
**Creatinine (mg/dL)**	1.22 ± 0.57	0.83 ± 0.05	1.79 ± 0.89	0.238
**Bilirubin (mg/dL)**	0.77 ± 0.16	0.83 ± 0.18	0.69 ± 0.12	0.523
**Albumin (g/dL)**	43.57 ± 0.93	42.95 ± 1.02	44.44 ± 0.74	0.251
**Prothrombin ratio (%)**	94.06 ± 4.25	94.25 ± 3.46	93.79 ± 5.18	0.940

*AST* aspartate aminotransferase, *ALT* alanine aminotransferase, *GGT* gamma glutaryltransaminase, *GT* genotype, *SOF* sofosbuvir, *LDV* ledipasvir, *2D* ritanovir boosted ombitasvir/paritaprevir, *3D* ritonavir boosted ombitasvir/paritaprevir/dasabuvir, *BMI* body mass index; *HCV* hepatitis C virus, *HIV* human immunodeficiency virus, *IVDA* intravenous drug abuse, *MSM* men who have sex with men, *GT* genotype

The majority of patients were male (72.2%), the main route of transmission was intravenous drug use (61.1%) and HCV-GT1 was most common (69.4%). Approximately one third (35.3%) of patients was treatment-experienced. The proportion of treatment-experienced patients was not significantly higher in the 2D/3D group (46.7% vs 28.6%; *p* = 0.462). Except for one patient, all patients (97.2%) were on ART prior to and during treatment and in the majority of cases (84.4%) HIV-RNA was suppressed (<50 copies/ml). The distribution of fibrosis stages assessed by transient elastography was as follows when using cut-offs proposed by Castera et al. [30]: F2 (7.1–9.4 kPa): *n* = 16 (44.4%), F3 (9.5–12.4 kPa): *n* = 5 (13.9%), and F4 (≥12.5 kPa): *n* = 5 (13.9%). Of the patients 6 (16.6%) had portal hypertension as indicated by a hepatic venous pressure gradient ≥6 mm Hg.

No statistically significant differences in patient characteristics were observed when comparing SOF/LDV and 2D/3D patients. The antiretroviral therapy used during SOF/LDV or 2D/3D treatment is shown in Table 2.

**Table 2:** Antiretroviral therapy

Treatment regimen	SOF/LDV	2D/3D
3TC	6 (28.6%)	7 (46.6%)
ABC	5 (23.8%)	7 (46.6%)
TDF	15 (71.4%)	8 (53.3%)
FTC	14 (66.7%)	8 (53.3%)
ATZ	1 (4.8%)	1 (6.7%)
DRV	2 (9.5%)	0
FPV	1 (4.8%)	0
RTV	4 (19.0%)	1 (6.7%)
RPV	1 (4.8%)	1 (6.7%)
ETR	2 (9.5%)	0
RAL	5 (23.8%)	3 (20%)
DTG	10 (47.6%)	10 (66.7%)

*3TC* lamivudine, *ABC* abacavir, *TDF* tenofovir disoproxil fumarate, *FTC* emtricitabine, *ATZ* atazanavir, *DRV* darunavir, *FPV* fosamprenavir, *RTV* ritonavir, *RPV* rilpivirine, *ETR* etravirine, *RAL* raltegravir, *DTG* dolutegravir

**Fig. 1:**
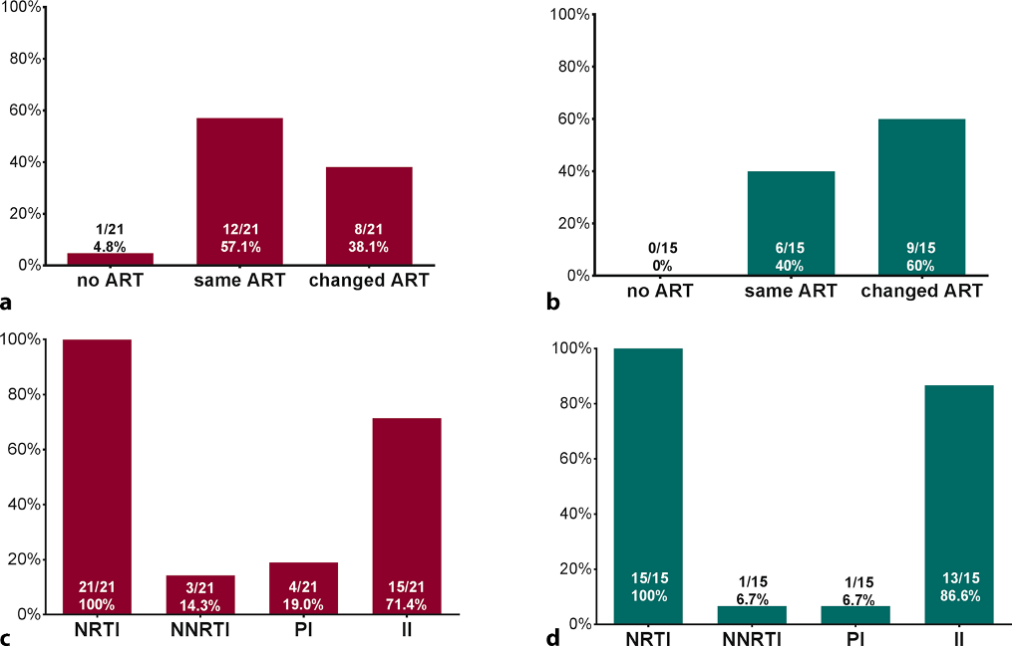
Antiretroviral therapy prior to and during anti-HCV therapy. **a,** **b** Percentage of patients without ART during therapy, or with same ART as before anti-HCV treatment, or with switched ART to a compatible ART regimen prior to therapy, indicated separately for SOF/LDV and 2D/3D. **c,** **d** Numbers and proportion of patients using different classes of ART drugs in their ART regimens during anti-HCV therapy, indicated for SOF/LDV and 2D/3D, respectively.* ART* antiretroviral therapy, *SOF* sofosbuvir, *LDV* ledipasvir, *2D* ritonavir boosted ombitasvir/paritaprevir, *3D* ritonavir boosted ombitasvir/paritaprevir/dasabuvir, *NRTI* nucleoside reverse transcriptase inhibitor, *NNRTI* non-nucleoside reverse transcriptase inhibitor, *PI* protease inhibitor, *II* integrase inhibitor

The majority of patients in both groups received nucleoside reverse transcriptase inhibitors (NRTI) combined with an integrase inhibitor (II). In 8 (38.1%) SOF/LDV (Fig. 1a) and 9 (60%) 2D/3D patients (Fig. 1b) ART had to be switched to a regimen containing 2 NRTI and an II prior to HCV therapy. In one patient, without HIV therapy at baseline who had a preserved CD4+ T‑cell count, ART was initiated at week 12 of anti-HCV therapy.

In the SOF/LDV group, the NRTI backbone was combined with NNRTI in *n* = 3 (14.3%), PIs in *n* = 4 (19.0%), and with IIs in *n* = 15 (71.4%) patients (Fig. 1c). In the 2D/3D group, IIs were more common with *n* = 13 (86.6%), while only *n* = 1 (6.7%) patient received NNRTIs and PIs, respectively (Fig. 1d).

TDF (SOF/LDV: 15 [71.4%], 2D/3D: 8 [53.3%]) and emtricitabine (SOF/LDV: 14 [66.7%], 2D/3D: 8 [53.3%]) were the most commonly used NRTIs, while dolutegravir (SOF/LDV: 10 [47.6%], 2D/3D: 10 [66.7%]) was the most commonly used II.

**Fig. 2:**
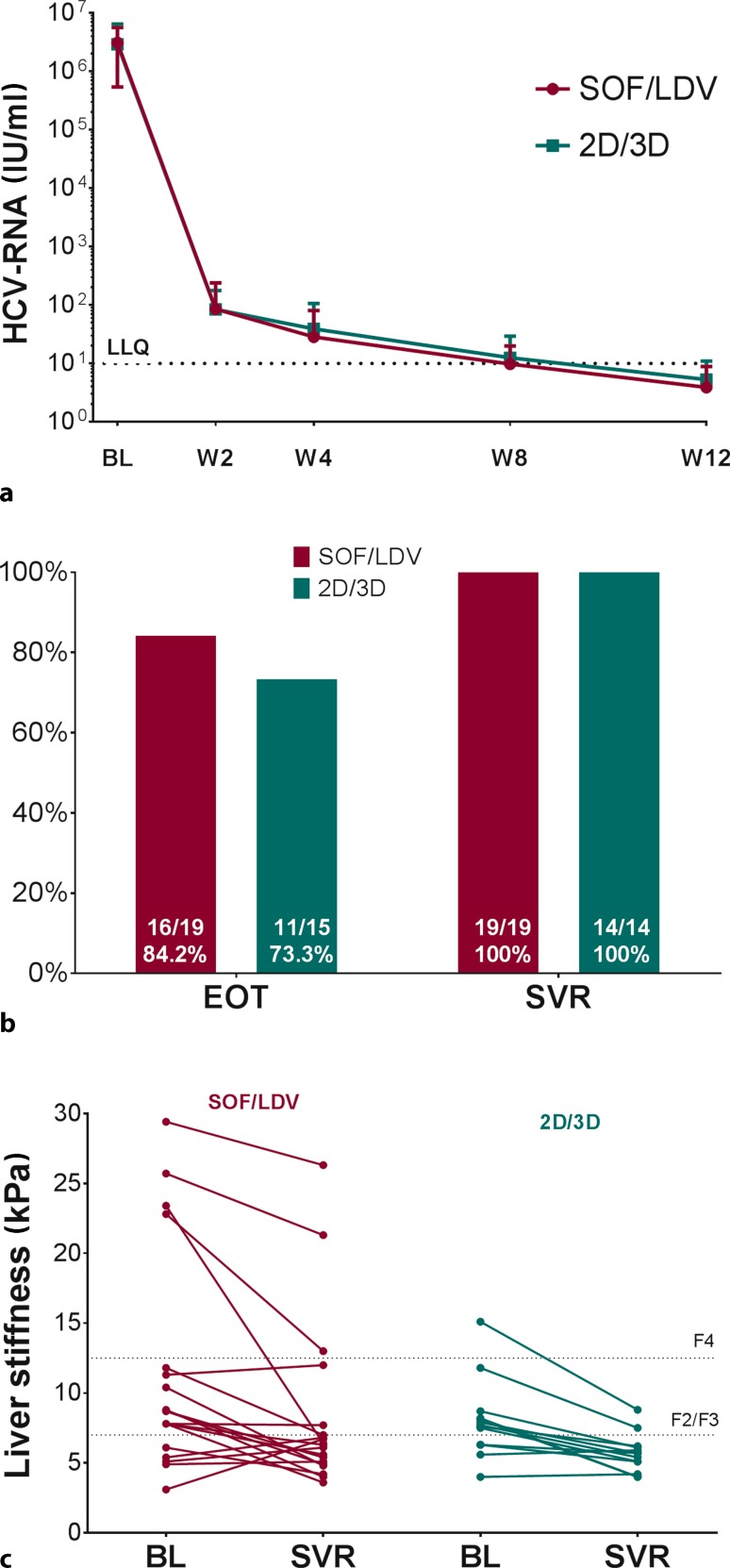
Treatment response. **a** Viral kinetics of HCV-RNA at baseline and during therapy (weeks 2 to 12) are shown as mean ± standard error of the mean at the respective time points for SOF/LDV and 2D/3D, respectively. **b** Proportion of patients with end of treatment negativity and SVR after cessation of therapy are shown for SOF/LDV and 2D/3D, respectively. **c** Changes in liver stiffness from baseline to follow-up (evaluated at SVR) are depicted for SOF/LDV and for 2D/3D patients, respectively. *SOF* sofosbuvir, *LDV* ledipasvir, *2D* ritonavir boosted ombitasvir/paritaprevir, *3D* ritonavir boosted ombitasvir/paritaprevir/dasabuvir, *BL* baseline, *W* treatment week, *EOT* end of treatment, *SVR* sustained virologic response, *TND* target not detectable

The virological response to SOF/LDV and 2D/3D regimens is depicted in Fig. 2.

The viral kinetics during SOF/LDV and 2D/3D treatment was similar. After 4 weeks of treatment 2 out of 18 (11%) and 9 out of 18 (50%) patients treated with SOF/LDV had undetectable HCV-RNA and HCV-RNA below the lower limit of quantification (LLQ) respectively, compared with 4/14 (28.6%) and 3/14 (21.4%). treated with 2D/3D. At treatment week 8 the same applied to 7/19 (36.8%) and 10/19 (52.6%) patients treated with SOF/LDV and 7 (46.7%) and 5/15 (33.3%) patients treated with 2D/3D (Fig. 2a). Treatment was prolonged for up to 24 weeks in 7 (33.3%) and 2 (13.3%) patients treated with SOF/LDV and 2D/3D, respectively.

In the SOF/LDV group 16 out of 19 (84.2%) patients had undetectable HCV-RNA at the end of treatment and 19 out of 19 (100% [95% CI: 80.2–100%]) patients achieved SVR. We observed no relapse or breakthrough, but two patients died during therapy from non-treatment-related causes and were excluded from the analysis. In contrast in the 2D/3D group 11 out of 15 (73.3%) patients had an end of treatment response but all 2D/3D patients (14 out of 14, 100% [95% CI: 74.9–100%]) went on to achieve SVR. One patient treated with 2D/3D was lost to follow-up and excluded from the analysis (Fig. 2b).

### Safety

The SOF/LDV and 2D/3D regimens were generally well-tolerated; however, one patient treated with SOF/LDV discontinued treatment at week 12 due to worsening of a pre-existing cardiomyopathy. Furthermore, two patients died from non-treatment-related causes: one due to a pre-existing CNS lymphoma, while the other death was AIDS-related. No patients treated with 2D/3D discontinued antiviral therapy prior to week 12 of 2D/3D.

### Change in liver stiffness

Paired liver stiffness measurements were available in 19 (90.5%) and 13 (86.7%) of SOF/LDV and 2D/3D patients, respectively. Between baseline and follow-up, liver stiffness decreased from 11.4 to 8.3 kPa (*p* = 0.008) and from 8.1 to 5.7 kPa (*p* = 0.001) in SOF/LDV and 2D/3D patients, respectively (Fig. 2c). Interestingly a small group of 5 (26.3%) and 2 (15.4%) patients showed increases in liver stiffness after SOF/LDV and 2D/3D treatment, respectively.

### HIV suppression during therapy

**Fig. 3:**
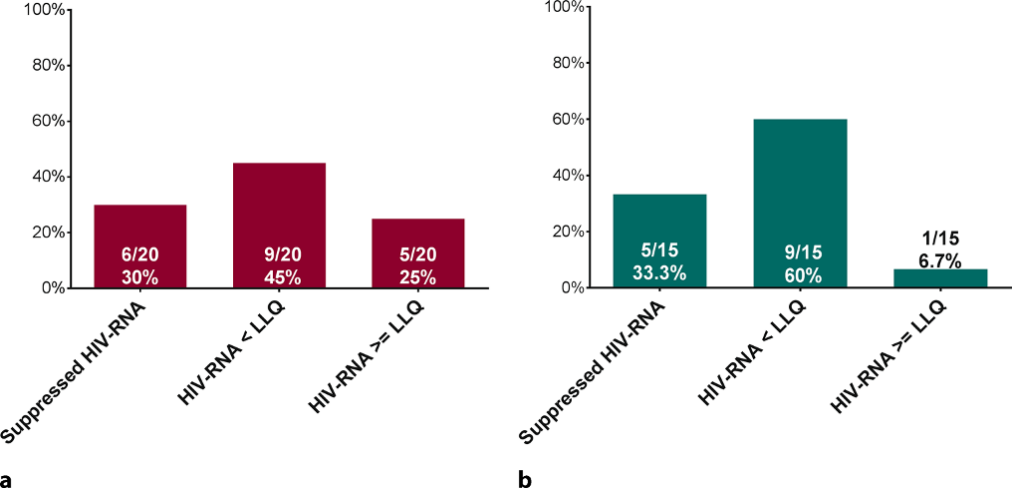
HIV suppression during anti-HCV therapy. Proportion of patients with continuous complete suppression of HIV-RNA during anti-HCV treatment, or with detectable but not quantifiable HIV-RNA levels <LLQ, or quantifiable HIV-RNA ≥LLQ during anti-HCV with SOF/LDV (**a**) or with 2D/3D (**b**). *LLQ* lower limit of quantification, *SOF* sofosbuvir, *LDV* ledipasvir, *2D* ritonavir boosted ombitasvir/paritaprevir, *3D* ritonavir boosted ombitasvir/paritaprevir/dasabuvir

Low HIV viremia (either HIV-RNA <LLQ or ≥LLQ) was common during anti-HCV treatment. In the SOF/LDV group, 9 (45%) and 5 (25%) patients showed detectable HIV-RNA <LLQ and HIV-RNA ≥LLQ, respectively (Fig. 3a). The patient without ART at baseline was excluded from this analysis. In the 2D/3D group, HIV-RNA ≥LLQ was less common with 1 (6.7%) and 9 (60%) showing detectable HIV-RNA <LLQ (Fig. 3b); however, the trend toward a higher rate of HIV-RNA ≥LLQ in the SOF/LDV group was not statistically significant (*p* = 0.207). Prior to treatment 7 (35%) and 3 (15%) patients showed detectable HIV-RNA <LLQ and HIV-RNA ≥LLQ in the SOF/LDV group, respectively. In the 2D/3D group 5 (33.3%) and 1 (6.7%) patients showed detectable HIV-RNA <LLQ and HIV-RNA ≥LLQ prior to treatment, respectively. The difference in viremia prior to and during DAA treatment was not statistically significant between the SOF/LDV and the 2D/3D groups.

## Discussion

Our study aimed to investigate the real-life efficacy of SOF/LDV and the 2D/3D regimen in a cohort of thoroughly documented HIV/HCV coinfected patients. Due to the reimbursement requirements of the Austrian health insurance, nearly all patients had to have at least liver fibrosis ≥F2. Nevertheless, compared with other studies [31], cirrhosis was less common as most patients with an urgent need for therapy have been treated as soon as sofosbuvir/daclatasvir became available [28].

The ION-4 [17] and the TURQUOISE-1 [18] trial assessed the efficacy of SOF/LDV and 3D in HIV/HCV coinfected patients and demonstrated remarkable SVR rates of ≥95%; however, their generalizability to the real world has recently been questioned [21]; therefore, we investigated whether SVR rates ≥95% were achievable in a real-world setting. In analogy to other reports [31], our study confirms these numbers; however, another recent study by Lakshmi et al. reported a substantially lower cure rate of only 83.3% in their HIV/HCV coinfected cohort [32]. We observed an intent to treat SVR rate of 100% (95% CI: 80.2–100%) and 100% (95% CI: 74.9–100%) in patients treated with SOF/LDV and 2D/3D, respectively. Two patients died during therapy from a non-treatment-related cause and were excluded from the analysis. Additionally, several studies in HCV monoinfected patients with HCV-GT1 achieved equally high SVR rates of ≥90% [33–36]; however, only 75% of genotype 3 patients were cured [34]. Our particularly high SVR rate of 100% among patients with HCV-GT3 might be explained by the low proportion of patients with cirrhosis and longer treatment durations.

Notably, since RBV reduces quality of life by inducing anemia [37] we abstained from prescribing RBV. A recent review on anti-HCV therapy in patients with cirrhosis concluded that RBV confers no additional benefit in most patients as it only marginally increases SVR rates at the cost of increased adverse events [38]. Interestingly, 3 (14.3%) and 4 (26.7%) patients had detectable HCV-RNA at the end of treatment in the SOF/LDV and 2D/3D group, respectively. In all but one case, HCV-RNA was below the lower limit of quantification. All HIV/HCV coinfected patients with low but detectable HCV-RNA at the end of treatment achieved SVR. Low levels of viremia assessed by the Abbott RealTime HCV assay at later stages of treatment have previously been reported; however, this was deemed insignificant as it does not predict treatment failure [39]. Further studies are warranted to investigate the ideal treatment duration for every patient as it was done in the IFN era [40].

A change in antiretroviral therapy was necessary in the majority of patients receiving 2D/3D, as coadministration with NNRTIs and PIs is not recommended [23]; however, ART remained unchanged in two patients receiving atazanavir and rilpivirine. A recent study demonstrated that morning administration of atazanavir exhibits no clinically relevant DDIs [41]. Coadministration with rilpivirine was well-tolerated in healthy volunteers; however, this combination is not recommended as the observed elevations in rilpivirine levels raise the risk for AEs [42]. We chose a regimen consisting of 2 NRTIs and an II, most commonly a combination of tenofovir disoproxil fumarate, emtricitabine and dolutegravir. Due to expected DDIs or potential for DDIs, ART was changed in about 40% of patients receiving SOF/LDV. In retrospect, this might not have been necessary in most cases [23].

Only a minority of patients had an undetectable HIV-RNA throughout anti-HCV treatment. We observed a high number of HIV-RNA blips, defined as HIV-RNA <LLQ; however, there was no difference in the amount of viremia and blips in the clinical visits prior to and during DAA treatment. Since HIV-RNA blips are a common finding in HIV-infected patients [43], they should not be over-interpreted as an indicator for clinically relevant DDIs. Nevertheless, we cannot exclude that minor DDIs contributed to the frequency of low levels of detectable HIV viremia despite ART during concomitant anti-HCV regimens.

A recent publication by Sulkowski raised the question on whether HIV/HCV coinfected patients should still be considered a special population in the era of second generation DAAs, since SVR rates are similar to HCV monoinfected patients. Notably, he concluded that the term is still warranted as this population faces specific challenges including reinfection, frequent drug interactions and the unanswered question of shortened treatment durations [44]. While the term ‘hard to cure’ is not justified anymore, we agree that treating HIV/HCV coinfected patients in specialized centres is still a necessity to address these clinical challenges.

Similar to previous studies [27, 28] we observed a rapid decline in liver stiffness which is unlikely to be explained by fibrosis regression due to the short time frame. The exact nature of how HCV eradication reduces liver stiffness remains unknown, yet possible mechanisms were discussed in a previous study [28]. The observed improvements are not limited to liver stiffness but also include effects on portal pressure. Previous studies observed a significant reduction of portal hypertension after SVR to IFN-based [45] and IFN-free therapies [46, 47]*.*

The European Association for the Study of the Liver (EASL) recommends treatment of HIV/HCV coinfected patients regardless of degree of fibrosis especially in those at risk of transmitting HCV [9]. Historically, in Austria the majority of HIV/HCV coinfected patients remained untreated [10]. With the approval of second generation DAAs the reason of under-treatment has shifted from medical contraindications and patient refusal to an economic dilemma. Although treatment of patients with mild fibrosis but at high transmission risk is cost-effective [48], the Austrian health insurance still employs strict criteria for reimbursement to stick to the budget. Among 163 HIV/HCV coinfected patients who were referred to the HIV & Liver Outpatient Clinic at the Medical University of Vienna for evaluation of antiviral therapy, 26 viremic HIV/HCV coinfected patients are now ‘waiting’ until liver disease progresses to fibrosis stage F2 in order to become eligible for reimbursement of IFN-free DAA therapy.

In summary, we achieved excellent SVR rates of 100% in a real-life cohort of HIV/HCV coinfected patients treated with SOF/LDV and 2D/3D. The ART was changed prior to 2D/3D and SOF/LDV treatment in a significant proportion of patients in order to avoid expected DDIs. The HIV-RNA blips during DAA treatment are common but should not be over-interpreted as clinically significant DDIs. The HIV/HCV coinfected patients still represent a special population as DDIs, as well as the prevention and management of reinfections requires treatment at specialized centers. Despite excellent SVR rates in clinical practice and the EASL recommendation to prioritize treatment for HIV/HCV coinfected patients regardless of fibrosis, this is not yet the case in Austria. Thus, a large number of patients at our outpatient clinic remain untreated even though highly effective IFN-free DAA treatments are available.
